# The Chicago Octopus

**Published:** 1905-06

**Authors:** 


### THE TEXAS MEDICAL JOURNAL.

AUSTIN, TEXAS.

A MONTHLY JOURNAL OF MEDICINE AND SURGERY.

EDITED AND PUBLISHED BY
F. E. DANIEL, M. D.
Mrs. F. E. DANIEL, Business Manager.

Published Monthly at Austin, Texas. Subscription price $1.00 a year in advance.

### THE CHICAGO OCTOPUS:
### The Journal American Medical Association.*

ITS EDITOR, ITS AIMS AND ITS METHODS.

*New Converts Shout Loudest.*

**Ye Editor.**

The present editor of the great (so called) organ of the American Medical Association, I have always understood, is a reformed homœopath, who practiced on an exclusive (and absurd) dogma twenty years. If this be true, it will account for much in the policy and course of the publication, which, unfortunately for the mass of the profession, he directs, on the principle that new convert shouts loudest. In no other way can his newly awakened fanatacism on medical ethics be accounted for. "Their sectarian

*Independent Medical Journals please copy.

title makes them quacks," says the old code, which code has been abandoned for the milder "principles of ethics" since his accession to the mantle of Father Davis and the great John B. Hamilton (by the bye, it is much too large for him and swallows him as a peck measure would a pea). His zeal in the cause of "ethics," which he has so recently espoused, leads him to extremes. He would out-Herod Herod. He has instigated and brought about the creation of a commission or committee of censors who promulgate the order that proprietary medicines are unethical, and unless the proprietors will surrender for publication the secrets of their business, make common property of the formulæ for making their products, so that every substitutor can make and sell "something just as good," they are to tabooed, and denied space in the pure and undefiled pages of the great "ethical" organ, ye Octopus! Great Cæsar! The sword of Democles was not half so terrible. Pity, isn't it?

This ridiculous and impossible policy, I am sure, does not represent the wishes and views of the medical profession of America, of which 85 per cent are not members of the American Medical Association, and, unless a broader and more representative and popular man be put in the editorial chair, and becomes the director of the business affairs of the Association, never will be. It does not represent the wishes of the 15 per cent who have been coaxed, bull-dozed or otherwise induced to join the Association. I'll give an instance in support of this assertion:

The American Congress on Tuberculosis was the first body organized to fight tuberculosis. It was organized in 1900, and held its fourth annual session in St. Louis in October, 1904, under the auspices and upon invitation of the World's Fair management, and the patronage and authority of the Federal government. Secretary of State Hay invited delegates to be sent from all foreign countries. The meeting was successful, largely attended, and gave the first impetus to the movement now so general, enlisting influence that will be effective in limiting the spread of consumption. It was participated in by many of the most distinguished physicians from Canada and Nova Scotia, Mexico, Central America, South America, Cuba and Porto Rico and of the South and West; Illinois, the home of the Octopus, being largely represented, prominent members of the A. M. A. and readers of the great Octopus. Dr. Simmons, ye editor, devoted one inch of the valuable space at his disposal to a sneer at the "so-called Congress on Tuberculosis." Common decency, or, at least, a decent regard for his readers, many of whom are members of the Congress, working earnestly in the cause of humanity and the public health, demanded the support and approval of the great so-called "representative organ" of the great A. M. A., and the neglect of it was so clearly the result of personal bias and prejudice towards the organizers of the Congress (the Medico-Legal Society) on the part of ye narrow editor as to excite disgust.

Thus has the *Journal of the American Medical Association* aroused antagonism in the ranks of the profession, when a broader, more liberal and sensible course would have cemented the friendship of the members and built up the Association. Not only has antagonism in the ranks been aroused, but a large element of the medical press is arrayed in hostility against it; and the proscription of the high-class pharmacal products, along with the nostrums in the newspapers, no discrimination except on impossible and outrageous terms, has aroused the animosity of the powerful proprietary interest, driven them to organize for defense, and set in motion influences which may lead to the withdrawal of patronage altogether from that organ, and perhaps all medical journals.

**Ye Aims.**

The Medical Association of Texas, under the old organization, crumbled constantly. It was building up and falling down, so that, from 1884 to 1903 inclusive, it gained only 100 members and represented only 6 per cent of the profession of the State. We joyfully hailed a plan of reorganization that seemed to remedy this, and secure cohesion; hence, we all worked for it with a vim, co-operating with the *paid organizer* (paid by the Octopus), without a suspicion of the ulterior and nefarious object aimed at by that great Devil-fish, which, in the light of recent developments, is revealed. Why should the A. M. A. pay to have the State associations organized? It was an investment. Every member whipped in is $5.00 for the Octopus. The Octopus has corralled the State associations, and, through its tentacles (State Association journals), is, like its prototype, making them feeders to its rapacious jaws. Its object is to *crush out* all individually owned medical journals that have the courage to criticise it; and, co-operating with the patent-medicine interest, with which (the *American Medical Journalist* says) it has made a "combine" and an agreement for co-operation to suppress such proprietary medicines as will not make common property of their formulæ. The The *American Medical Journalist* adduces evidence of such an agreement. (See March and April numbers of that publication.) If there is a shadow of truth in this charge, it is enough to excite derisive laughter and provoke indignation and utter contempt for a publication which, while posing as the representative organ of the medical profession of America, and champion of medical ethics, stands in with the promoters and exploiters of the worst nostrums on the market, the newspaper catarrh and syphilis and lost-manhood specifics. The *Reporter*, the journal of the North America Retail Druggists Association (so the *American Medical Journalist* says) gleefully points out that the less proprietary medicines prescribed by doctors the more patent medicines they will sell, and the profit is better. They have a systematic rake off from these patent medicines, and they urge all druggists to "Push Miles," "Push Peruna," "Push Swamp Root," etc. They (the J. N. A. R. D. Assn.) smack their lips in anticipation of the success of the Octopus in forcing doctors to quit prescribing the legitimate proprietary articles, and says that then the doctors will write more prescriptions, on which there is a larger rake off.

In brief, I conceive the aim of the Octopus to be, in addition to the scheme above pointed out, the establishment of a great medical journal trust, and, already, many of the State Associations have fallen into the trap and are applying the screws. Well, you will see that they will have their eyes opened perhaps when it is too late; and what a falling off, my countrymen, there will be! When the rich and grasping Octopus shall have accomplished its nefarious purpose, and has the State associations "grabbed," ye scheming editor can say, as did he of Khorassan: "Ye would be dupes and ye are."

The better class of proprietary medicines are well known to the physicians who read, and they are no more "nostrums" than my horse. "Noster" means "ours." My horse is "ours,"—the medicines are not. The principal and active ingredients are well known, and the dose. It is not necessary that the prescriber should know the adjuvants and non-active ingredients, any more than he should bear in mind the exact composition and amounts of each ingredient, say of the C. C. Pills of the Dispensatory, and I'll bet an apple ye editor of ye Octopus, nor his "council" can not tell you, off-hand, what they are made of. Why should a proprietary medicine, often a household necessity, be any more "unethical" than any other trade-marked article, say, Sapolio, or Uneeda Biscuit, or Royal Baking Powder? Or than surgical instruments, all of which are protected. Bosh!

Whipping the Devil Around the Stump.—It is a case of do as I say, and not as I do, with ye Octopus; for ye Octopus, in every issue, violates the rules it has laid down for its satellites, the State journals. I give below two of those rules, enough to show the absurdity of the demand and the evasion of the same by ye managing editor of the Octopus. In every issue there appear advertisements without the pretense of a formula, and some formulæ are given which might as well be Greek, as far as conveying any information goes; and other ads. appear which specifically state what diseases they will "cure." An instance of the first mentioned I give here: "* * * Antipyretic, anti-neuralgic and hypnotic. A derivative of antipyrin, in which an H atom of the pyrozolon group is replaced by a *dimethylamido* group." That's as clear as mud. Here is another: "Polantin, patented in Germany, the United States, England, etc. Testimonials from 86 physicians, 326 cases." (No formula.) "Benolgur-Capsules." (Formula: *Capsulæ Dipterocarpi Benzoinatæ* B. C. C." (Clearer than mud.) Another: "Unguentine." No formula, but the statement is published that "it has been endorsed and used by the medical profession for many years." Does Dr. Simmons know what unguentine is made of? Oh, consistency, thy name is not Simmons, by a jug full. The *Journal of the California Medical Association* is constantly calling on the Octopus to purge its pages or drop the mask of "ethics," and the *Charlotte Medical Journal* says: "The whole cause of the bad ethics so often seen scattered through the reading matter and advertising pages of the journal is due to Dr. Simmons, and not to the trustees of the Association. He is a cunning politician and a typical commercial gentleman. As long as he has control of the Association journal it will succeed financially, but will never take a decent stand ethically."

Here are two of the rules governing the admission of advertisements in ye Octopus, and consequently in its satellites, the State journals:

"Rule 1.—No article will be admitted unless its active medicinal ingredients and the amounts of such ingredients, in a given quantity of the article, be furnished for publication."

"Rule 4.—No article will be admitted whose label, package or circular accompanying the package contains the names of diseases, in the treatment of which the article is indicated. The therapeutic indications, properties and doses may be stated."

The manufacture of prescriptions by wholesale, by learned, honest and capable men to fill an active demand by the medical profession, is a *distinct advance in medical science,* a convenience and an economy.

I say the use of such articles as are advertised in the "Red Back" is ethical, wise, right and proper. I know the vile stuff that is advertised in the papers, and they couldn't get in on any terms. Such manufacturers and importers as are represented in my pages are public benefactors, and are the ethical physician's friends and allies.

**Ye Methods.**

The methods inaugurated by ye Octopus may be summed up in few words: Bull-dozing, intimidation, threats of ostracism, denunciation as unethical, exclusion from list of ethical preparations, exclusion of the 85 per cent of doctors from "Who's Who." In short, Trades Unionism!

The "Red Back" has ever been the champion and defender of medical ethics, which we understand means pure morals, right living, consideration for others and the rights of others. This, of course, embraces charity, tolerance, right and justice. There is a vast difference between ethics and fanaticism. I want to say here, that I do not consider the use by physicians of the better class of proprietary medcines made for and advertised solely to the medical profession as a breach of the Principles of Ethics adopted by the American Medical Association. See Section 8. On the contrary, it is right, proper, and necessary. It is convenient and economical. Such articles are not "secret medicines," "nostrums," in any sense. Every physician knows in a general way what they are composed of and the dose. Our profession claims to be eclectic, broad and comprehensive, and claims the right to use anything and everything for the cure or alleviation of disease, material or immaterial, that experience has shown to be beneficial; and the practice of medicine is, essentially, empirical, in the sense that our knowledge is gained by experience. Who knows how drugs "cure"? For years the use of quinine was entirely empirical. It had been observed in thousands of cases that it would cure chills, but how? A physician is justified in using anything that his judgment dictates, and which he knows from experience, or the experience of others, will benefit his patient. The dictum from headquarters that he shall not prescribe proprietary medicines is high-handed, dictatorial and an insult to his intelligence; and the assertion that he lets the pharmacist think for him is the veriest tommy-rot. The Octopus wants to think for him.

Will the vast body of American physicians be thus "like dumb cattle driven"? by a fanatical howler for "ethics," which every issue of the Octopus violates according to the standard he has himself established? Or will they demand his removal, and that one of the Davis or Hamilton kind be seated on the tripod, who will make the *Journal A. M. A.* what it formerly was, and not the laughing stock for many good men, the vehicle of coercion and intimidation, through which our new convert may vent his spleen and dislikes, by sneering at any movement for the betterment of man, that does not originate with Him.

---

THE "Red Back" completes its twentieth year with this number,—under one management, the founder. It enters upon the twenty-first round strong, vigorous and splendidly supported by the best men in Texas, and a clean and cash-paying advertising patronage, much of which has been with me twenty consecutive years! That tells the tale. Its policy will be as it ever has been—to fearlessly condemn quackery and shenanegan, and to advocate and defend that which I think is right,—the champion always of high and honorable professional character. It is personally owned and is independent in all things and neutral in nothing that touches the welfare of the legitimate medical profession.

I make my bow to my old guard who have stuck to me and the "Red Back" through evil as well as good report, and ask all who read this to speak a good word for the game cock and assist it in its new boom and laudable work. Selah!

